# Utility of 18F-FDG PET with a Semi-Quantitative Index in the Detection of Sarcomatous Transformation in Patients with Neurofibromatosis Type 1

**DOI:** 10.1371/journal.pone.0085954

**Published:** 2014-02-06

**Authors:** Patrick Combemale, Laurence Valeyrie-Allanore, Francesco Giammarile, Stephane Pinson, Bernard Guillot, Denis Mariano Goulart, Pierre Wolkenstein, Jean Yves Blay, Thomas Mognetti

**Affiliations:** 1 Rhône-Alpes multidisciplinary Center for the treatment of Neurofibromatosis type 1, Léon Bérard Comprehensive Cancer Center, Lyon, France; 2 National reference center for neurofibromatosis type 1, Department of Dermatology, Henri Mondor Hospital, UPEC, Créteil, France; 3 Department of Nuclear Medicine, Léon Bérard Comprehensive Cancer Center, Lyon, France; 4 Languedoc multidisciplinary Center for the treatment of Neurofibromatosis type 1, Saint Eloi University Hospital, Montpellier, France; 5 Department of Nuclear Medicine, Saint Eloi University Hospital, Montpellier, France; 6 National reference center for sarcoma, Léon Bérard Comprehensive Cancer Center, Lyon, France; University of Verona, Italy

## Abstract

**Background:**

Malignant peripheral nerve sheath tumors (MPNSTs) are a serious complications of neurofibromatosis type 1 associated with poor prognosis and deeper lesions can be difficult to diagnose. 18-FDG PET improves the detection of malignancies. However, the criteria for malignancy, notably the SUVmax threshold, are not standardized. Therefore, the aim of the study was to evaluate a semi-quantitative index for the reproducible detection of MPNST with FDG PET.

**Methods:**

It is a multicenter retrospective study conducted between 2000 to 2012. All patients with NF1 referred for suspected MPNST underwent PET. Since SUVmax was not available until 2004 in our centers, we had to settle for the semi-quantitative method used at that time, the uptake ratio between the tumor and the normal liver (T/L ratio) with 1.5 as the cut-off for malignancy. When dedicated PET with SUVmax became available, the semi-quantitative analysis of PET images remained, along with SUVmax.

**Results:**

113 patients with 145 tumors were included. PET assessment revealed 65 suspected lesions with T/L >1.5 and among these, 40 were MPNSTs. 80 tumors were classified as non-suspicious, and 79 were benign. The 1.5 T/L cut-off had a negative predictive value (NPV) of 98,8% and a positive predictive value of 61,5%. The positive likelihood ratio (LR) was 4,059, the negative LR was 0,032 with 97% sensitivity and 76% specificity.

**Conclusions:**

This study, which is among the largest published, confirms the utility of PET for detecting NF1-associated MPNSTs. A semi-quantitative index, the T/L ratio with a cut-off of 1.5, allowed sensitive and specific differentiation of malignant from benign tumors better than SUVmax. When T/L was <1.5, MPNSTs were ruled out with 98,8% NPV. When T/L was >1.5, there was a strong suspicion of malignancy. This semi-quantitative analytical method is as simple as SUVmax, but is more sensitive, more reproducible and non-user-dependent.

## Introduction

Neurofibromatosis type 1 (NF1) is one of the most common autosomal dominant genetic disorders, with an incidence of 1/2500 live births [1]. One of its most serious complications is the development of malignant peripheral nerve sheath tumors (MPNST), which are also known as neurofibrosarcomas. The estimated risk of developing MPNST is 1.6/1000 NF1 cases annually and 8 to 13% across a lifetime [2]. MPNST is one of the most frequent causes of death in NF1 patients, together with brain cancer and vascular disease [3]. Late diagnosis of MPNST is associated with poor prognosis [4]. Surgical biopsy or excision is recommended for patients with superficial involvement. For those with deeper lesions, the surgical approach is sometimes difficult, as the suspected tumor is embedded among other non-malignant neurofibroma masses and is not easily recognizable. Therefore, techniques that allow non invasive exploration of the lesion can justify and/or guide surgery or microbiopsy. The current standard imaging modality is magnetic resonance imaging (MRI), which has limited specificity and sensitivity in sarcoma. Over the past few years, tumor evaluation by ^18^F-FDG positron emission tomography (FDG PET/CT) has significantly improved the detection and monitoring of malignancies, including soft tissue sarcomas. Elevated SUV_max_ values are significantly correlated with malignancy [5]; however, the use of SUV_max_ as a gold standard is controversial because the threshold for malignancy is not standardized and significant technical variations have been observed among different medical teams and even among patients [6]–[8].

Therefore, the aim of the present study was to test the value of another index, the T/L SUV_max_ ratio, and its reproducibility for the detection of MPNST with PET/CT [9], [10].

## Materials and Methods

### Study population

The study population was composed of all patients referred to four NF1 reference centers (Rhône-Alpes, Languedoc Roussillon, West of France and Paris) with suspected malignant transformation. A total of 113 patients (63 men, 50 women, ratio 1.26) were retrospectively included. The median age was 31.3±17.1 years (range 2–77 years). Diagnosis was made using the National Institutes of Health (NIH) criteria [11]. NF1 was confirmed when patients met two or more of the seven criteria described by the NIH (Table 1).

**Table 1:** Consensus criteria for the diagnosis of neurofibromatosis type 1.

NIH criteria for the diagnosis of neurofibromatosis type 1	
1	Six or more cafe-au-lait skin macules >5 mm in prepubertal individuals and >15 mm in postpubertal individuals
2	Two or more neurofibromas of any type or one plexiform neurofibroma
3	Axillary or inguinal freckling
4	Two or more Lisch nodules
5	Optic glioma
6	Bone lesion with sphenoid dysplasia or thinning of the long bone cortex with or without pseudarthrosis
7	A first-degree relative (parent, sibling, or offspring) that meets NIH criteria

This study includes 38 patients from a preliminary publication in 2007 [12].

### Study design

This retrospective study was conducted between October 2000 and July 2012. All consecutive patients with one or more of the following criteria for suspicion of malignant involvement [13] were eligible for the study: presence of a growing and/or cystic mass (neurofibroma or plexiform NF) with or without pain, severe prolonged pain unresponsive to standard treatment, occurrence or aggravation of neurological symptoms (neurological pain, sensory-motor deficit, dysphonia, dysphagia), general physical deterioration, standard radiological results (osteolysis), and imaging-based evidence of malignancy (by MRI, CT or ultrasound). The study was performed according to French laws at the time of the initiation of the study and followed the principles laid down in the Declaration of Helsinki. Oral consent was obtained after a complete explanation by the patients or legal representatives for minors, and it was reported in the patient’s medical record. Formal approval as a written consent was not specifically needed, as it was waived by the IRB of the institution (Centre Leon Berard), which approved this retrospective study.

### 18F-FDG PET/CT

All referred patients underwent FDG-PET because FDG was indicated for tumor detection, although not specifically for MPNST detection.

Dedicated PET/CTs were not commonplace in 2000 when our first patients underwent imaging. Coincidence Detection Emission Tomography (CDET) was more widely available. Later, PET equipment evolved and we collected PET studies performed on a variety of cameras: a Marconi Irix (a CDET camera) until 2004, and then a Philips Gemini Dual, a Philips Gemini GXL, a Philips Gemini TF16 PET-CT, and a Siemens Biograph 4. Imaging procedures were very consistent among centers: patients were at rest for at least six hours prior to examination, plasma glucose levels were measured before scintigraphy, and images were acquired 60 minutes after injection of 200–500 MBq ^18^F-FDG (IBA, France). Uptake of the tracer was assessed on axial, coronal and sagittal images.

Since SUV_max_ cannot be measured on CDET cameras (because they do not correct attenuation), the nuclear medicine physicians had to use a semi-quantitative method, the uptake ratio between the tumor and the normal liver (T/L ratio) and a T/L ratio of 1.5 as the cut-off for malignancy There was little alternative option for another uptake reference, hence the liver reference. It appears that they initially set the T/L ratio SUV_max_ cut-off for risk of malignancy at 1, as described in the literature [14]. Then, as the number of false positives was high, with serious consequences for patients (systematic use of surgical resection or biopsy), it became obvious the threshold had to be revised and they raised it to 1.5. The rationale for this is not known but they consistently continued to use semi-quantitative analysis of PET images along with SUV_max_ when imaging was performed on a dedicated PET.

### Patient management and follow-up assessment

Thereafter, only tumors with T/L SUV_max_ ≥1.5 were considered suspicious, and such patients were referred for surgery or biopsy. In contrast, patients with non-suspicious lesions (T/L SUV_max_ <1.5) were assigned to clinical monitoring only, possibly including PET/CT, with visits every three months for at least 12 months. Surgical resections were performed in some cases of major functional impairment, multidisciplinary team recommendation, or at the patient’s request. At each investigating center, pathological material was reviewed by the same pathologist with expertise in sarcoma. Immunological analysis was performed using S100 protein, anti-CD34, anti-Ki67, and anti-claudin 1 antibodies. Tumors were graded according to the criteria established by the French Federation of Comprehensive Cancer Centers (FNCLCC).

### Statistical analysis

PET/CT assessment and the standard procedure (surgical biopsy and clinical monitoring) were compared for sensitivity, specificity, positive and negative predictive values (PPV and NPV), and positive and negative likelihood ratios (PLR and NLR). The distribution of T/L ratios between benign and malignant tumors was assessed using a non-parametric test (Mann-Whitney test). Only p-values <0.05 were considered statistically significant. All statistical analyses were performed with SPSS v.17 (IBM).

## Results

A total of 113 patients with 145 lesions were included in the study (Table 2). None of the patients had a previous history of MPNST.

**Table 2:** Demographic and baseline characteristics of patients.

Variables	n (%)
Sex (n = 113)	
Male	63 (55.8)
Female	50 (44.2)
Lesions (n = 145)	
Lumbar pelvic area	48 (33.1)
Lower limb	35 (24.1)
Cervical and facial area	28 (19.3)
Upper limbs	19 (13.1)
Thorax	12 (8.3)
Missing	3 (2.1)
Symptoms (n = 146)	
Pain	60 (41.1)
Pain and tumor growth	40 (27.4)
Pain and neurological deficit	12 (8.2)
Asymptomatic growth	22 (15.1)
Functional impairment	3 (2.1)
Asymptomatic	9 (6.2)

Symptoms for inclusion were pain (n = 60, 41%), pain associated with tumor growth (n = 40, 28%), pain with neurological deficit (n = 12, 8.3%), asymptomatic growth of a neurofibroma mass (n = 22, 15%), functional impairment such as cough or vascular compression (n = 3, 2%), or asymptomatic tumor discovered while evaluating a symptomatic tumor (n = 9, 6.2%).

Among the 145 tumors, 80 had a T/L ratio SUV_max_ <1.5. Of these, 79 were confirmed as benign neurofibromas (by either pathology (n = 55) or follow-up assessment from 3 to 10 years, (n = 24)) and one was malignant, corresponding to a 98.8% NPV.

**Figure 1:**
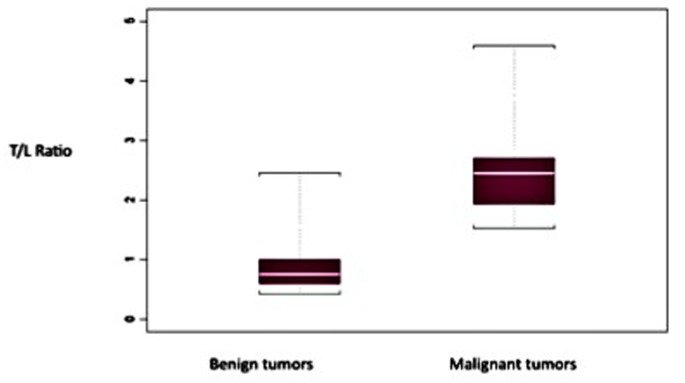
Mean T/L ratio in benign and malignant lesions.

Concerning the 65 other lesions (with a T/L ratio ≥1.5), 40 were identified as MPNSTs and 25 as benign neurofibromas, corresponding to a 61.5% PPV. The PLR (equal to sensitivity/1-specificity)) was 4,059 (1/[1–0.75]; 95% CI: 2,874–5,731), and the NLR (equal to 1-sensitivity/specificity) was 0.032 (CI 95%: 0,005–0,223) with 97% sensitivity and 76% specificity (figure 1). Threshold defined using a ROC curve for the specific sensibility, specificity values is 1.48.

Incidentally, we did not find any significant correlation between malignancy and absolute tumor SUV_max_ among the subset of patients studied with dedicated PET/CT.

## Discussion

MPNSTs represent about 10% of all soft tissue sarcomas, and are associated with NF1 in 32 to 60% of cases [15], [16]. Moreover, they are one of the major causes of death in NF1 patients. The relative risk of MPNST in these patients is high (RR: 113) and is increased in patients receiving radiation therapy [17]. These tumors usually occur earlier in NF1 than in the general population (30 to 32.5 years *vs*. 60 years) [4]. Tumors are more frequently diagnosed in patients with deep NF involvement. In a series of 120 MPNSTs, Ducatman *et al*. found that 81% of cases had a history of neurofibroma [18], and particularly internal plexiform neurofibroma, with a relative risk of 20 for sarcoma [19]. The most common sites of MPNSTs are the roots of the limbs, the extremities and the retroperitoneal space [20], as observed in our study. Clinical symptoms suggestive of malignant transformation include neurological deficit, rapid enlargement of pre-existing tumors and increasing pain that is unresponsive to symptomatic treatment [13]. However, tumor enlargement is the indicator with the highest PPV (92%) and NPV (95%) values. Nevertheless, these symptoms may not be specific to malignant disease [20], as confirmed by the present study. In fact, among the 40 patients with the most specific association, pain and tumor swelling, malignant transformation was observed in 43% of cases. In comparison, presence of pain alone was associated with MPNST in 28% of cases. Nevertheless, the combination of pain and tumor enlargement remains significantly predictive of MPNST in 17/40 of our cases (43%), and should continue to be of interest in clinical practice.

MPNSTs associated with NF1 are of higher histological grade than those in the general population [21] and display poorer prognosis and earlier metastatic spread. The survival rate at five years is also lower (21% *vs.* 35%) compared with patients with sarcoma in the general population (68%) [21]–[23]. This poor prognosis could be partly explained by delayed diagnosis. Early diagnosis is essential in order to improve outcomes [17]. This can be achieved by histological analysis, which might be relatively easy for superficial tumors, but is more difficult in patients with deep involvement. Surgical access may be difficult or even risky when the tumor is deeply located (i.e., in the mediastinum or the pelvis). In this context, the estimated risk of generating uninformative results is approximately 5% [24].

CT and MRI images can be useful to detect MPNSTs and many criteria have been proposed for these methods [25]. De Schepper *et al.* suggested that tumor diameter >66 mm, no abnormal signal intensity on T2-weighted images and the presence of a heterogeneous signal intensity on T1-weighted images, combined with neurovascular deficit and >50% tissue necrosis, are suggestive of malignant transformation [26]. Early contrast enhancement (<6 sec.) had 91% sensitivity and 72% specificity. More recently, Van Herendael *et al*. reported that intermuscular distribution, location on the course of a large nerve, nodular morphology, and overall non-homogeneity on T1- and T2-weighted images and on T1-weighted images after gadolinium contrast injection were all factors significantly associated with malignancy (p<0.05) [27]. However, the target sign (the central area of low signal intensity surrounded by a rim of high signal intensity) is not specific [28]. Therefore, although it provides some interesting information, MRI is not sensitive enough (50–80%) for the diagnosis of malignant tumors [29]. Moreover, the presence of multiple tumors on a given site makes MRI assessment even more difficult.

PET/CT can be used to assess the whole-body volumetric distribution of biological tracer molecules labeled with positron-emitting isotopes. The radiotracer ^18^F-FDG has been validated for the measurement of local glucose uptake and is routinely used in the evaluation and follow-up of many tumor types. Thus, ^18^F-FDG tissue distribution 60 minutes after injection reflects the rate of glycolysis, and malignant areas can be identified due to their increased glycolytic activity and over-expression of glucose transporters [30]–[32].

SUV and its derived parameters (e.g. SUV_max_ ) are quantitative rather than semi-quantitative measurements. SUV_max_ determination involves measuring the absolute FDG concentration through PET, which is adjusted for weight and injected activity. SUV is a unitless value and can be calculated using the following equation: SUV = PET tissue concentration (MBq/kg)/(injected activity (MBq)/body weight (kg)).

Analysis of PET-CT images is mostly based on simple visual analysis and SUV_max_. Even though qualitative visual analyses are fast and reliable, the results are highly operator-dependent in borderline situations. Since many non-cancerous lesions have the potential to accumulate glucose, SUV_max_ does not constitute definitive proof of malignancy. Moreover, it is well known that SUV_max_ presents limitations and can be influenced by many individual (e.g., time between injection and PET, blood glucose concentration, body composition, kidney elimination) and physical (e.g., reconstruction algorithms, resolution recovery, spillover, image noise) variables. Due to these potentially complicating factors, which cannot be measured or normalized, SUV_max_ has often been criticized based on limited reproducibility [7], [8], [33].

PET/CT assessment has good diagnostic accuracy for many cancers [30]–[35]. Warbey *et al*. [36] showed that delayed acquisition (four hours after FDG administration) is beneficial when assessment is based on SUV_max_, since FDG uptake continues to rise until that point. However, because of the 110 min half-life of this costly tracer and the pressure for patient throughput, a large majority of PET/CT centers routinely acquire images at one hour.

A meta-analysis of patients with soft tissue and bone sarcomas published in 2004 [6] reported 91% sensitivity, 85% specificity and 88% efficacy using the SUV_max_ based approach. Moreover, PET/CT can predict the histological grade of soft tissue sarcomas as a function of SUV_max_. Several studies have explored the role of this method in the diagnosis of MPNSTs and have confirmed its interest for the detection of MPNST [38]–[42]. However, the very heterogeneity of their results makes it difficult to draw a single conclusion. For example, Cardona *et al.* [43] reported 100% sensitivity and 83% specificity in a population of 25 patients (of whom five had NF1) with an absolute SUV_max_ cut-off value for malignancy of 1.8. But other recent studies have demonstrated that an absolute SUV_max_ >3 is associated with poor prognosis and is correlated with Ki67 levels [41], [42], [44]; Tsai [45] set the SUV_max_ malignancy threshold at 4. Karabatsou also reached a 4 SUV_max_ cut-off, but this was in a very small set of 9 patients [46]. In 40 patients, including 16 MPNST, Benz [38], set the SUV_max_ cut-off at 6.1, with 94% sensitivity and 91% specificity. Finally, several works by Ferner’s group [47], [48] confirmed the diagnostic efficacy of PET/CT in the detection of MPNST but also the high variability of the SUV_max_ cut-off values proposed to distinguish between MPNST and benign tumors. In 2000, the authors set the malignancy threshold at 2.5 while noticing one false negative among 28 patients with 7 MPNST [47]. Then, in 2008, in a series of 105 patients (including 116 tumors, 29 MPNSTs), sensitivity in diagnosing NF1-associated MPNST was 89% and specificity 95% [48]. Their results showed that a high SUV_max_ level is significantly correlated with malignancy, with an absolute SUV_max_ cut-off of 3.5, but that there were no malignant tumors when the SUV_max_ was below <2.5. Moreover, there is a wide overlap between these 2 values, which should be reviewed clinically. These results were confirmed by Warbey [36] one year later, but the same group obtained similar results (97% sensitivity and 87% specificity). The authors found a cutoff of malignancy of SUV_max_ >2.35 (60% specificity) with early imaging at 90 min and at 3.1 for delayed imaging at 240 min (100% sensitivity, 76,6% specificity). However, they concluded that using a cut-off of 3.5 on delayed imaging had the maximal sensitivity for malignancy and showed that there is a correlation between mean maximum SUV_max_ and tumor grade [36].

Nevertheless, false positives were also reported, and the method proved unable to accurately distinguish between malignant and benign lesions [48].

The heterogeneity of the proposed SUV_max_ cut-offs for the detection of MPNSTs suggests that absolute SUV_max_ based analysis has poor reliability. This is relevant given its known limitations regarding reproducibility [7], [8], [33]. In addition, different groups reported contradictory results, making comparisons difficult.

Therefore, other authors have performed semi-quantitative analysis, considering not SUV_max_ but a ratio between tumor and safe tissue SUV_max_ [31]. Miyamoto *et al*. [37] showed that semi-quantitative analysis was effective for the diagnosis of orbital tumors, with significantly higher tumor/controlateral normal tissue values in malignant than in benign lesions. Although little used, semi-quantitative analysis has been suggested by various authors, with the most used reference being the liver [49]. Authors have compared SUV_max_ and tumor/liver (T/L) ratios in non-secreting adrenal tumours. In addition to a good correlation, they showed that a T/L ratio with a threshold of 1.8 could determine malignancy with a 100% PPV and a 91% NPV [9], [10]. This threshold was identical in two different teams, suggesting good reproducibility among users [9], [10]. In contrast, Mantaka used the T/L ratio SUV_max_ for detecting liver’s tumors, and found a cut-off for malignancy of 1 [14].

In our department, the initial threshold was empirically set at 1, but apparently resulted in a number of false positives, and was therefore empirically raised to 1.5, with no more false positives (including retrospectively patients first evaluated with a cut-off for malignancy of 1) [12]. The T/L ratio was significantly higher (2.5) in proved malignancies than in benign samples (p<0.002). However, an elevated absolute SUV_max_ (>2) was not found to be statistically associated with malignancy.

To our knowledge, ours is the only study on the detection of MPNSTs with PET/CT that is not based only on absolute tumor SUV_max_ assessment. Our use of the T/L uptake ratio, although somewhat empirical, appears to be reliable, reproducible and less subject to SUV_max_ variability (possibly because semi-quantitative assessment bypasses some of the factors influencing FDG uptake) [49].

Significantly, this multicenter study demonstrates that this ratio is independent of the medical team and the PET/CT camera. This type of analysis is just as easy to perform and can even be done retrospectively if all DICOM images are archived.

We were able to determine that a 1.5 cut-off provides a 98.8% NPV and 61.5% PPV. Our only false-negative finding could not be explained by low tumor aggressiveness since the patient’s tumor was grade 1. Shahid *et al*. also reported a false-negative patient with a grade 3 tumor [50]. Unfortunately, our data did not enable us to identify a correlation between false positivity and dysplasia in plexiform neurofibromas. Such tumors have a higher risk of malignant transformation, but a recent study showed that plexiform neurofibromas have similar uptake, whether or not they are associated with dysplasia [51]. This point should be examined in future studies.

This retrospective, multicenter study is among the largest to explore the utility of PET/CT in NF1 patients. Our findings show that PET/CT can be used to identify deep MPNSTs. Moreover, we have identified simple, reproducible, non-user-dependent PET/CT criteria for ruling out malignancy, which is essential for the effective use of this technology. We show that a seldom used semi-quantitative analysis, with a T/L ratio cut-off of 1.5, has a NPV of 98.8%, with an acceptable PPV of 61.5%. To our knowledge, no study has provided such promising results based on absolute SUV_max_ values. These results are a strong argument in favor of this tool. Patients with a T/L ratio <1.5 can safely be assigned to a simple monitoring strategy, thus avoiding unnecessary risky or mutilating surgery. Patients with a T/L ratio ≥1.5 require pathologic sampling before surgery is decided upon. However, false positive results may be obtained, and medico-surgical confirmation is required before any potentially detrimental therapeutic decision is made.

Because our method can also be used retrospectively, we encourage other authors to match their existing series against it to confirm the reproducibility of our results.
